# Visualization of Phototherapy Evolution by Optical Imaging

**DOI:** 10.3390/molecules28103992

**Published:** 2023-05-09

**Authors:** Zhiheng Li, Zheng Li, Jie Wang

**Affiliations:** 1 College of Materials and Chemical Engineering, Zhengzhou University of Light Industry, Zhengzhou 450002, China; lizhiheng@zzuli.edu.cn; 2 Wuhan Academy of Agricultural Sciences, Wuhan 430072, China; lizheng_hubeiwuhan@foxmail.com; 3 The Key Lab of Health Chemistry & Molecular Diagnosis of Suzhou, College of Chemistry, Chemical Engineering & Materials Science, Soochow University, Suzhou 215123, China

**Keywords:** photodynamic therapy, photothermal therapy, fluorescence, imaging, cancer

## Abstract

Phototherapy, including photodynamic therapy (PDT) and photothermal therapy (PTT), is a non-invasive and effective approach used for cancer treatment, in which phototherapeutic agents are irradiated with an appropriate light source to produce cytotoxic reactive oxygen species (ROS) or heat to ablate cancer cells. Unfortunately, traditional phototherapy lacks a facile imaging method to monitor the therapeutic process and efficiency in real time, usually leading to severe side effects due to high levels of ROS and hyperthermia. To realize precise cancer treatment methods, it is highly desired to develop phototherapeutic agents possessing an imaging ability to evaluate the therapeutic process and efficacy in real time during cancer phototherapy. Recently, a series of self-reporting phototherapeutic agents were reported to monitor PDT and PTT processes by combining optical imaging technologies with phototherapy. Due to the real-time feedback provided by optical imaging technology, therapeutic responses or dynamic changes in the tumor microenvironment could be evaluated in a timely manner, thereby achieving personalized precision treatment and minimizing toxic side effects. In this review, we focus on the advances in the development of self-reporting phototherapeutic agents for a cancer phototherapy evaluation based on optical imaging technology to realize precision cancer treatments. Additionally, we propose the current challenges and future directions of self-reporting agents for precision medicine.

## 1. Introduction

Cancer, as one of the diseases with the highest mortality rate, is, at present, the leading cause of deaths worldwide, and has thus become a serious health threat in modern society [1,2]. The early diagnosis of and precise therapy for cancer are highly significant to human health. At present, surgery, chemotherapy, and radiotherapy are the most commonly used methods to treat cancer in clinical applications [3,4]. Although these therapies can kill cancer cells effectively, they still face formidable challenges. For example, surgery cannot remove the tumors completely due to the unrecognized edge of the tumor tissue, which usually leads to tumor recrudesce [5]. Chemotherapy and radiotherapy are usually conducted with an overdose of drugs and excessive radiation to achieve better treatment effects, which results in serious side effects and damage to the surrounding healthy tissues [6,7]. Additionally, the risk of multidrug resistance of tumors and tumor metastasis may increase [8]. Therefore, it is highly desirable to develop advanced, efficient, and selective therapies to improve the therapeutic efficiency and reduce the undesired side effects.

With the rapid development of nanomedicine, many novel cancer therapies have been presented and attracted great attention in recent years, such as photo-, immune-, gene, chemodynamic, and various synergistic therapies [9,10]. Among them, phototherapy, including photodynamic therapy (PDT) and photothermal therapy (PTT), emerges as a non-invasive cancer treatment method thanks to its precise spatiotemporal controlled ablation toward cancer cells [11]. The principle of PDT and PTT is that a photosensitizer (PS) or PTT agent is activated with appropriate light irradiation to produce cytotoxic reactive oxygen species (ROS) or heat to ablate cancer cells [12]. However, the high level of ROS and hyperthermia caused by superfluous agents and high-power light irradiation increases the risk of severe side effects on normal tissues near tumors [13,14]. To solve these problems, the therapeutic process and efficacy of PDT and PTT should be monitored and evaluated in real time by imaging technology. Through the feedback of imaging, the therapy process could be personalized with high selectivity and minimized side effects by optimizing the doses of agents, irradiation time, and laser-power density [15,16].

Volumetric measurement and biomarker detection are the two usually used methods to monitor and evaluate cancer therapeutic effects [17,18]. The measurement of tumor-size changes is a visualized method that can be achieved by using traditional biomedical imaging technologies, including magnetic resonance imaging, computed tomography, and positron emission tomography [19]. However, volumetric measurement always requires several weeks or months post-treatment to evaluate the treatment outcomes by measuring the obvious shrinkage of tumors [20]. Since it fails to monitor the early treatment effect, the overall course and efficiency of the treatment, as well as the cure rate of the patients, may be influenced [18]. Biomarker detection, including cytological and histopathological analyses, usually relies on a biopsy, which is a partial and invasive method [21]. Thus, it is unsuitable for continuous and real-time monitoring methods. Therefore, it is highly desired to design non-invasive, in situ, and real-time evaluation methods for cancer therapy to realize precise and efficient cancer therapy methods. 

**Figure 1:**
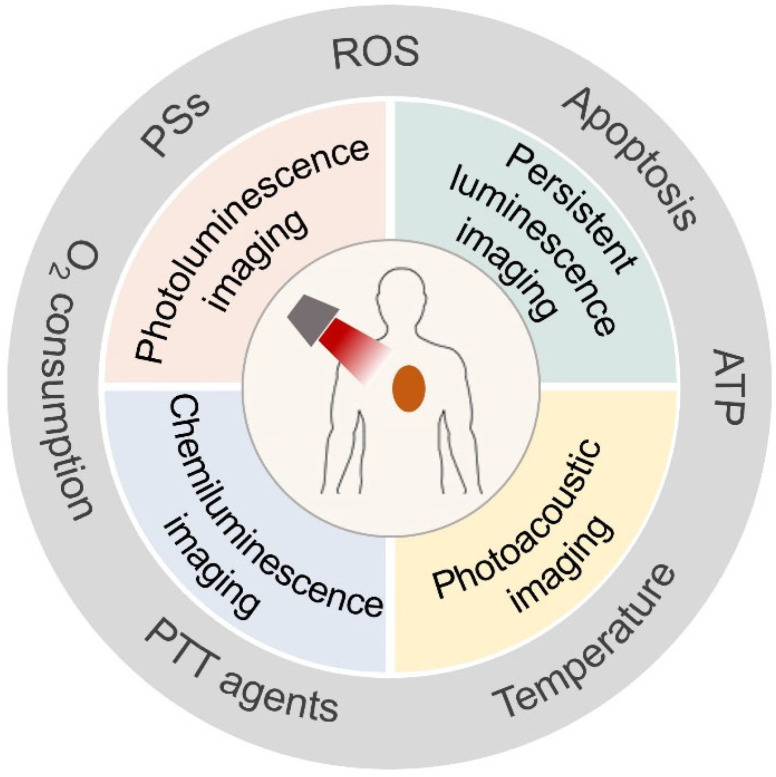
Optical imaging used for phototherapy evaluation.

As a convenient, rapid, and non-invasive strategy, optical imaging can be employed to report the dynamic changes in tumors during PDT and PTT processes for the early evaluation of the therapeutic efficacy [11]. Recently, a series of self-reporting PS and PTT agents with luminescence properties were developed to monitor PDT and PTT processes [16]. The luminescence intensity of these self-reporting PS and PTT agents was positively correlated with therapeutic parameters, including the release of PDT/PTT agents, ROS production, temperature, and cell apoptosis, which makes it possible to evaluate the therapeutic effect in real time and non-invasively. Due to the self-monitoring feature of optical imaging, the irradiation time and intensity of the light source, as well as the doses of PS and PTT agents, can be accurately controlled to achieve the accurate and effective treatment of tumors with negligible side effects. In this review, we showcase the advancements achieved, to date, in the past 5 years in the development of self-reporting PS and PTT agents for evaluating cancer phototherapy based on optical imaging technologies (Figure 1). A brief introduction of cancer PDT and PTT and the optical imaging methods is provided, including photoluminescence, persistent luminescence, chemiluminescence, and photoacoustic imaging techniques. We further present the performance of self-reporting phototherapeutic agents sensitive to the release of PS and PTT agents, ROS generation, heat, cell apoptosis, and other related biomarkers for monitoring PDT and PTT processes. Finally, we discuss the challenges and future perspectives of self-reporting approaches for accurate and efficient phototherapy treatments.

## 2. Phototherapy

The use of light for disease treatment dates back to the 19th century [11]. A representative example of phototherapy was the use of short-wavelength ultraviolet light to treat lupus vulgaris in 1903, which was realized by Nobel Prize winner in Physiology or Medicine, Niels Finsen [22]. Since then, modern phototherapy has gradually developed. After decades, hyperbilirubinemia was treated using blue light, and millions of infants were cured [23]. Presently, many therapeutic devices based on laser treatment are fabricated and applied in clinical practice. At present, phototherapy is widely used in the treatment of various diseases, such as psoriasis, atopic dermatitis [24], acne vulgaris [25], vitiligo [26], bacterial infection [27], and cancer [28]. Considerable efforts made in nanotechnology have promoted the development of cancer phototherapy, including PDT and PTT, which is a non-invasive and effective approach for tumor suppression using phototherapeutic agents with light irradiation [29]. The following section introduces the mechanisms and processes of PDT and PTT.

### 2.1. PDT

**Figure 2:**
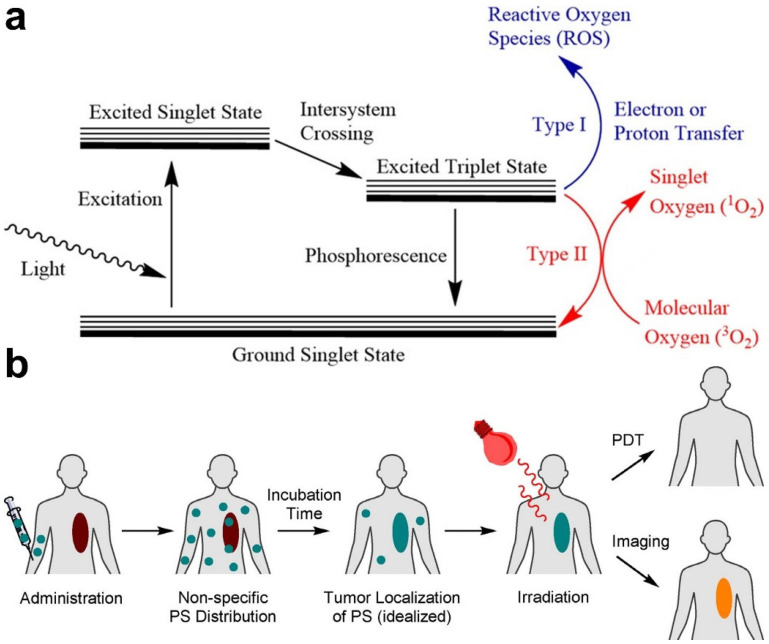
(**a**) Photophysical mechanism of ROS generation in PDT. (**b**) Schematic illustration of a typical PDT process. Reprinted with permission from ref. [31]. Copyright 2022 WILEY-VCH.

PDT relies on three indispensable elements: PS, O_2_, and a light source [30]. With appropriate light irradiation, PS can be promoted to the excited state and transfers the absorbed energy to nearby O_2_ to generate ROS, especially singlet oxygen (^1^O_2_) through type-I or -II pathways (Figure 2a) [31]. ROS can damage cellular DNA and induce cell apoptosis to realize cancer PDT [30]. PDT was first conducted for cancer treatment by Dougherty et al. in the 1970s, who developed a variety of PS and light sources for the PDT of tumors [32]. Since the 1980s, more and more PSs have been synthesized and applied in clinical trials. At present, three generations of PSs have been developed [33]. The hematoporphyrin derivative was the most widely used first-generation PS for the treatment of lung cancer, bladder cancer, etc. [29]. To deepen the tissue penetration, second-generation PSs with near-infrared (NIR) absorption were designed, such as aminolevulinic acid and acridine orange [34]. Recently, in order to achieve greater therapeutic efficiency, first- and second-generation PSs were modified with multifunctional carriers, recognition units, or other functional groups to improve the selectivity and safety of PSs, which produce third-generation PSs [33]. With the study and development of third-generation PSs, cancer PDT with stronger effects and higher accuracy, as well as minimized side effects, were realized. 

In typical PDT treatments, PSs are first injected into the blood and circulated throughout the whole body. Then, PSs are selectively taken up by tumors and accumulate in cancer cells, rather than in normal tissues or cells. Subsequently, tumor tissues are exposed to light with a specific wavelength to activate the nontoxic PSs to generate ROS, mainly ^1^O_2_, which induces damage to cancer cells (Figure 2b) [29,31]. The PDT effect caused by ROS is achieved by inducing direct cytotoxic effects on cancer cells, including apoptosis, necrosis, or autophagy, destroying tumor vasculature, and creating inflammation accompanied by systemic immunity [35]. Furthermore, normal cells in the surrounding tissue avoid PDT damage because they are less sensitive to ROS. Clinical studies have shown that PDT is an efficient and safe strategy for many types of cancers [36].

### 2.2. PTT

**Figure 3:**
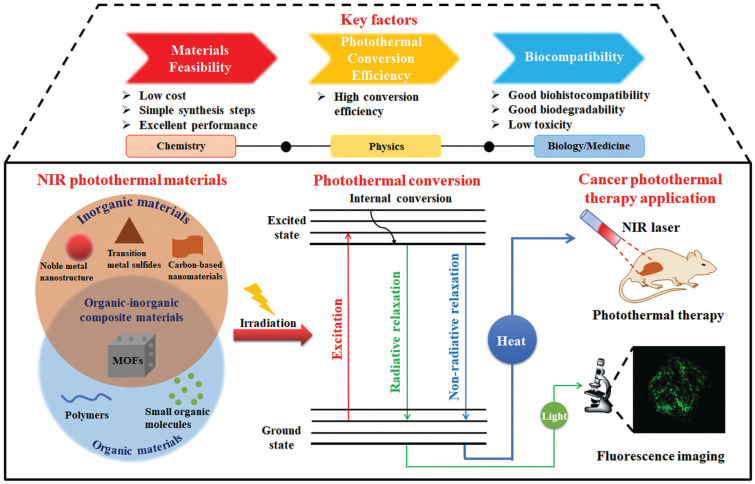
Schematic illustration of PTT agents and the photothermal conversion mechanism in the PTT process. Reprinted with permission from ref. [38]. Copyright 2021. The Royal Society of Chemistry.

PTT is a NIR-light-induced thermal therapeutic method and has caught researchers’ attention in recent years. In 2003, PTT for cancer therapy was first reported by Hirsch et al. [37]. In this pioneering work, gold colloid was coated on the surface of silica nanoparticles to obtain a PTT agent, and breast carcinoma cells treated with this PTT agent were efficiently killed upon NIR-light irradiation. PTT agents and NIR-light sources are the two necessary elements in PTT [29], which is different from PDT since O_2_ and ROS are both not involved. Thus, PTT is more suitable for the therapy of hypoxic tumors. The PTT effect largely depends on the performance of PTT agents, whereas the photothermal conversion and light-absorption ability are the main factors that determine the performance of PTT agents [38]. Since 2003, there has been tremendous interest in synthesizing PTT agents, and various organic and inorganic PTT agents have been developed for cancer therapy, including metal materials, carbon materials, semiconductor molecules, and organic molecules (Figure 3) [29]. For example, plasmonic gold nanostructures, graphene, iron oxide, metal chalcogenide, polypyrrole, and polydopamine with high light-absorptivity and photothermal conversion performances have been widely used as PTT agents to increase the local temperature in tumors for cancer treatment [11,39]. Presently, great efforts in the use of PTT agents are being made to enhance the tumor selectivity, biocompatibility, and photothermal conversion ability for improving their therapeutic effects.

Similar to PDT, PTT agents are injected and accumulate in tumor tissues. Upon irradiation with NIR light, PTT agents absorb photon energy and are activated. The activated PTT agents collide with the surrounding molecules to return to the ground state and release vibrational energy to produce heat (Figure 3) [38,40]. As a result, the energy of light is converted to local heat through a nonradiative relaxation process to elevate the temperature of tumor tissues. After the local temperature of tumors increases to 42 °C or higher, some thermolabile proteins in cancer cells denature and co-aggregate with aggregation-sensitive proteins, resulting in the inactivation of downstream pathways, the damage of chromatin, and the suppression of DNA repair activity for cell ablation [29,41]. PTT is also a non-invasive, tunable, and safe therapy to inhibit tumors, which has been applied to many kinds of cancer therapy.

PDT and PTT as monotherapies usually cannot completely ablate tumors. Thus, PDT or PTT is generally applied in combination with other therapies, such as chemotherapy, radiation therapy, and immunotherapy, to enhance the therapeutic effects [42].

## 3. Optical Imaging Technology

Optical imaging offers a convenient tool for visualizing multiple dynamic biological events and disease progression, which plays a crucial role in fundamental biomedical research and clinical practice [43]. Optical imaging can realize the non-invasive visualization of biological processes at the molecular level for disease diagnosis and image-guided therapy [44]. Compared with other imaging techniques, such as magnetic resonance imaging, computed tomography, and ultrasound imaging, optical imaging exhibits higher sensitivity and spatiotemporal resolution, as well as non-invasiveness [43]. Photoluminescence imaging has been used in clinical practice for guiding surgery [45]. However, photoluminescence imaging is often interfered by autofluorescence produced by endogenous chromophores during light excitation. To avoid autofluorescence interference, background-free imaging technologies, including NIR photoluminescence, persistent luminescence, chemiluminescence, and photoacoustic (PA) imaging techniques, have been developed. We discuss these four types of optical imaging technologies below.

### 3.1. Photoluminescence Imaging

Photoluminescence is a common phenomenon and present in many organic and inorganic materials [45]. In the photoluminescence process, luminescent materials produce luminescence under the excitation of an external light source [16]. According to the luminescence wavelength and mechanism, photoluminescence is usually segmented into three categories: visible, NIR-I, and NIR-II fluorescence methods [45]. Due to the poor tissue-penetration depth and autofluorescence interference of visible light, more and more research is focusing on NIR-I and NIR-II imaging to achieve a greater penetration depth and signal-to-noise ratios. Photoluminescence imaging is a convenient and cost-effective tool used to dynamically observe the biological processes for biological analysis, disease diagnosis, and therapeutic monitoring [46]. Photoluminescence shows great potential to establish a relationship between luminescence intensity and the therapeutic effects of PDT or PTT. Recently, many researchers designed various PS or PTT agents with photoluminescence properties to monitor in real time treatment effects by recording the luminescence signal.

### 3.2. Persistent Luminescence Imaging

**Figure 4:**
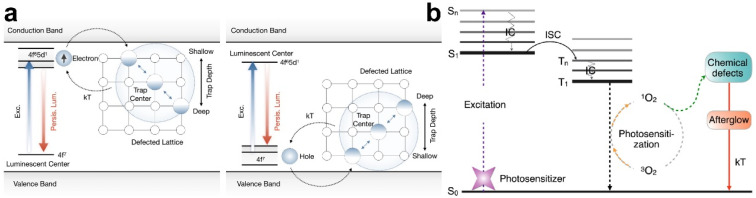
Schematic illustration of possible persistent luminescence mechanisms of inorganic (**a**) and organic (**b**) persistent luminescence materials. Reprinted with permission from ref. [51]. Copyright 2021. American Chemistry Society.

Unlike photoluminescence imaging, persistent luminescence imaging is a luminescent process that occurs after the termination of excitation light [47,48]. Due to the absence of an in situ excitation, tissue autofluorescence is effectively avoided and the influences of light scattering are also negligible [49,50]. Therefore, compared with photoluminescence imaging, persistent luminescence imaging possesses many advantages, including high imaging sensitivity and signal-to-noise ratio during an in vivo analysis [51]. Persistent luminescent materials can be divided into organic and inorganic materials, and they can act as an optical battery to temporarily trap excitation energy [51,52]. The slow release of trapped excitation energy produces persistent luminescence after the termination of excitation (Figure 4). Inorganic materials have high luminescence and persist for a long time; however, they suffer from producing potential toxic side effects, whereas recently developed inorganic materials show better biocompatibility but work for a shorter period of time. Although the properties of persistent luminescent materials still need to be optimized, they are potential candidates for the long-term monitoring of cancer therapy.

### 3.3. Chemiluminescence Imaging

Chemiluminescence imaging refers to the light-emission phenomenon induced by chemical reactions [45], which is a radiative relaxation and photon emission process of generated intermediates through reacting with reactive species [52]. Because of the absence of in situ light excitation, the background autofluorescence from biological tissues in chemiluminescence imaging is completely eliminated [44]. Thus, similar to persistent luminescence imaging, chemiluminescence imaging can also provide enhanced sensitivity and a high signal-to-noise ratio for biological imaging. At present, four kinds of chemiluminescent scaffolds have been developed, including luminol and its derivatives, peroxyoxalates, Cypridina luciferin analogs, and Schaap’s adamantylidene-1,2-dioxetane [53]. Most chemiluminescent scaffolds can only be activated to produce luminescence by single reactive species [51]. For example, luminol is activated by H_2_O_2_, and Cypridina luciferin analogs are activated by O_2_^−^. Owning to the reaction between chemiluminescent scaffolds and reactive species, such as ROS, chemiluminescence production can be monitored to evaluate the therapeutic efficacy of PDT.

### 3.4. Photoacoustic Imaging

**Figure 5:**
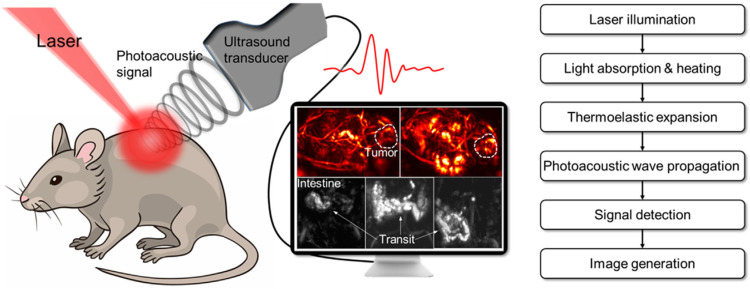
Schematic illustration of the principle of PA signal generation and image reconstruction during PA imaging. Reprinted with permission from ref. [54]. Copyright 2022. Elsevier B. V.

The poor penetration depth of optical probes is one of the challenges faced by optical imaging, even NIR-II lights. PA provides a deep tissue penetration of up to 7 cm and a higher spatial resolution [43]. During PA imaging, PA contrast agents absorb photo energy and convert it into heat, leading to an increase in sound waves induced by thermo-elastic expansion [45,46]. The sound waves can be detected by an ultrasound sensor and reconstructed into PA images (Figure 5) [54]. The ultrasonic signal produced by PA contrast agents is detected by an ultrasound transducer; thus, the background noise from tissues can be removed. Due to this unique effect, a variety of PA contrast agents, including inorganic nanomaterials (carbon nanotubes, gold nanoparticles, etc.), and organic small-molecule dyes have been developed for disease diagnosis and biomarker imaging [46]. At present, PA imaging has been applied in imaging-guided therapy, as well as the monitoring of the therapy process.

## 4. PDT Evaluation

With the purpose of promoting the accuracy and efficacy of PDT, many optical imaging probes have been developed to spatially and temporally monitor PDT-related parameters, including PS concentrations, ROS generation, cell apoptosis, and oxygen consumption. This section summarizes the recent advances in the evaluation of PDT efficacy according to different PDT-related parameters. Many of the developed methods for PDT efficacy monitoring are based on phosphors, including organic dyes, self-assembly molecules, polymers, covalent organic frameworks (COFs), metal-organic frameworks (MOFs), upconverting nanoparticles (UCNPs), and persistent luminescent nanoparticles.

### 4.1. Monitoring of PSs

**Figure 6:**
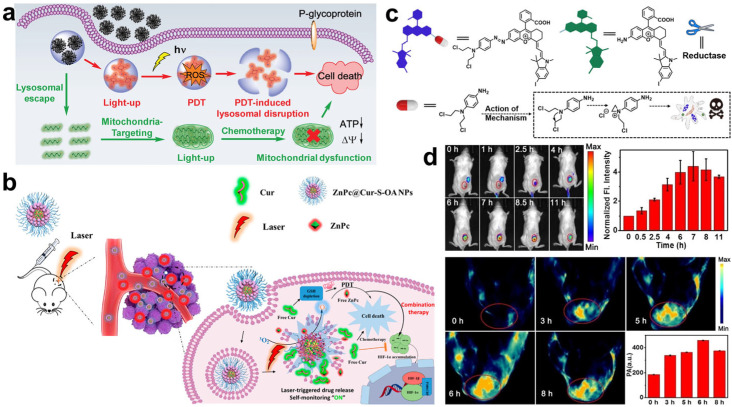
(**a**) Real-time monitoring of AlPcSNa_4_ and AIE-Mito-TPP release in cells for imaging-guided chemo-photodynamic therapy. Reprinted with permission from ref. [55]. Copyright 2018, WILEY-VCH. (**b**) ZnPc@Cur-S-OA nanoparticles used for self-monitored chemo-photodynamic therapy. Reprinted with permission from ref. [56]. Copyright 2020. American Chemistry Society. (**c**) Structure change and action mechanism of CS-P. (**d**) Fluorescence and PA imaging of 4T1 tumor-bearing mice after intravenous injection with CS-P. Reprinted with permission from ref. [57]. Copyright 2022, WILEY-VCH.

The visualization of PS release is of great significance for evaluating PDT performance and providing instructions for accurate PDT [16]. Recently, many PSs with photoluminescent properties have been designed and used as therapeutic drugs in PDT. The distribution of PSs in mice can be monitored by fluorescence imaging. The intensity of the fluorescence signal of PSs in tumors can reflect the concentration of PSs, as well as the yield of ROS, which indirectly reveals the effective dose of drugs. Ren et al. reported the preparation of therapeutic nanoparticles for synergistic chemo-photodynamic therapy through the self-assembly of PS AlPcSNa_4_ and chemotherapeutic drug AIE-Mito-TPP [55]. The release processes of AlPcSNa_4_ and AIE-Mito-TPP can be self-monitored by the fluorescence activities of AlPcSNa_4_ and AIE-Mito-TPP (Figure 6a). The fluorescence of AlPcSNa_4_ and AIE-Mito-TPP was quenched in the self-assembled nanoparticles. The nanoparticles were disassembled in the acidic environment upon internalization into cancer cells. As a consequence, the fluorescence values of AlPcSNa_4_ and AIE-Mito-TPP were recovered to produce red and green emissions, respectively. The accumulation of AlPcSNa_4_ and AIE-Mito-TPP in tumors was monitored in real time to achieve imaging-guided accurate cancer treatment. To further enhance the performance of PDT, Qu et al. used another chemotherapeutic drug, curcumin (Cur), to improve hypoxia and deplete GSH in tumors [56]. Similarly, ZnPc and Cur were self-assembled into ZnPc@Cur-S-OA nanoparticles for delivery. As shown in Figure 6b, the red fluorescence of ZnPc was monitored in real time to track the distribution of the nanoparticles in vivo, while the green fluorescence of Cur was switched on in tumors upon the dissociation of ZnPc@Cur-S-OA nanoparticles to monitor the release of ZnPc and Cur.

Some PSs were designed with PA properties, which were available to acquire feedback on PS release. Zhang et al. synthesized an NIR PS (CS-I) with both PA properties and NIR fluorescence [57]. CS-I was linked to a chemotherapeutic drug with a hypoxia-responsive azo bond to construct a prodrug CS-P (Figure 6c). The azo bond not only diminishes the fluorescence of CS-I, but can also inhibit the activity of the chemotherapeutic drug, which was proved by their singlet oxygen yields (ΦΔ). The ΦΔ of CS-P and CS-I were 0.16% and 7.4%, respectively, indicating that the ^1^O_2_ generation performance of CS-P was inhibited. After accumulating in tumor sites, the azo bond was destroyed in hypoxic tumors, leading to the release of CS-I and chemotherapeutic drugs. The release process was monitored in real time by PA and NIR fluorescence imaging techniques (Figure 6d). These examples show that PSs with fluorescence and PA properties exhibit great potential for precise cancer therapy.

### 4.2. Monitoring of ROS

**Figure 7:**
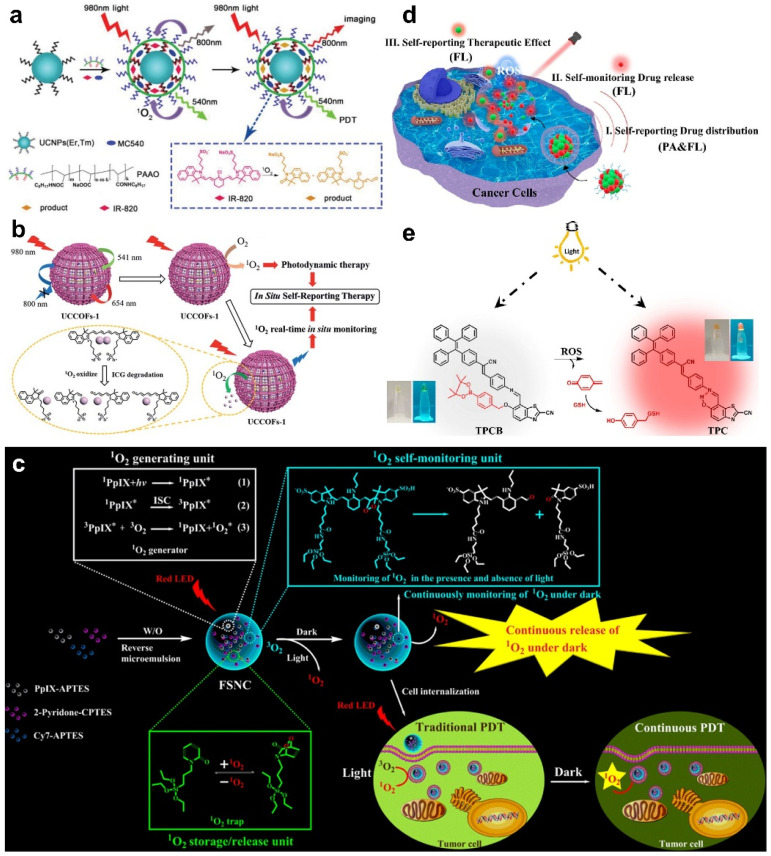
(**a**) Schematic illustration of the design of PAAO-UCNPs-MC540-IR820 and the ^1^O_2_-monitoring mechanism. Reprinted with permission from ref. [59]. Copyright 2019, WILEY-VCH. (**b**) Schematic illustration of the structure of UCCOFs and the mechanism of detecting ^1^O_2_ during PDT. Reprinted with permission from ref. [60]. Copyright 2019. The Royal Society of Chemistry. (**c**) Schematic illustration of multifunctional nanoplatform for PDT and ^1^O_2_ monitoring. Reprinted with permission from ref. [61]. Copyright 2019. American Chemistry Society. (**d**) Real-time monitoring of ZnPc@TPCB nanoplatform, ZnPc release, and ROS with PA and fluorescence imaging. (**e**) Structure of ROS probe TPCB and the structural conversion after the reaction with ROS. Reprinted with permission from ref. [62]. Copyright 2021. American Chemistry Society.

The exogenous ROS produced by PSs was the main performer to selectively suppress tumor growth, and the PDT effects were discovered to be correlated with the concentration of ROS, especially ^1^O_2_ [58]. Excessive doses of ROS may cause potential risks to patients, such as damage to normal tissues. Hence, the real-time monitoring of ROS generation in the PDT process is very important for evaluating the therapeutic effect and further determining the therapeutic parameters, so as to decrease undesired side effects. Recently, many researchers have reported that the Förster resonance energy transfer (FRET)-based UCNPs nanoplatform can realize PDT and ROS monitoring simultaneously. In Gu’s work, a UCNPs-based nanoplatform consisting of UCNPs, PS MC540, NIR dye IR-820, and an amphiphilic polymer PAAO was developed to monitor ^1^O_2_ production during PDT (Figure 7a) [59]. In this nanoplatform, the UCNPs emitted green and NIR lights at 540 and 800 nm, respectively. The emission of UCNPs at 540 nm could activate MC540 to produce ^1^O_2_, while the light at 800 nm was absorbed and quenched by IR-820. Due to the decomposition of IR-820 by ^1^O_2_, the light at 800 nm of UCNPs was continuously recovered. As a result, ^1^O_2_ production was directly detected by monitoring the luminescence intensity changes at 800 nm. The nanoplatform presented in this work not only produced ^1^O_2_ to achieve efficient tumor treatment, but it can also evaluate the dose–effect relationship between ^1^O_2_ and therapeutic efficacy in living systems. One year later, another FRET nanoplatform based on UCNPs and COF (termed UCCOFs) was constructed by Zhang et al. [60]. Indocyanine green (ICG) was immobilized on UCCOFs to detect the generation of ROS. The emission of UCNPs at 780 nm was quenched by ICG. The ^1^O_2_ produced by COF disintegrated ICG, which led to the continuous recovery of the luminescence at 780 nm (Figure 7b). The nanoplatform produced ^1^O_2_ for PDT and realized the real-time monitoring of ^1^O_2_ production in the therapeutic process at the same time, allowing efficient and precise cancer PDT in vivo.

Since the duration of light irradiation is usually short in the whole PDT process, the PDT process is generally intermittent, resulting in an unsatisfactory therapy effect. To overcome this limitation, Song et al. embedded PS, ^1^O_2_ storage and release units, and an ^1^O_2_ self-monitoring unit into a silica nanocarrier to construct a multifunctional nanoplatform that could simultaneously generate, store, release, and monitor ^1^O_2_ (Figure 7c) [61]. The ΦΔ of the nanoplatform was measured with a higher value compared to that of single PS and SiO_2_@PS, which might have been because the silica nanocarrier inhibited the quenching of ^1^O_2_ by solvent molecules and the continuous release of ^1^O_2_ in the dark. ^1^O_2_ was persistently produced by this nanoplatform for self-sustained PDT, and the release process of ^1^O_2_ was monitored in real time by observing the fluorescent bleaching behavior of the ^1^O_2_-monitoring unit. In order to increase the accuracy of PDT, the release processes of PS and ROS can be visualized simultaneously with dual-modality imaging. In Qu’s work, PSs ZnPc and aggregation-induced emission (AIE) ROS probe TPCB were self-assembled into a ZnPc@TPCB nanoplatform [62]. This self-assembled nanoplatform was non-photoactive, but exhibited strong PA signals for the real-time visualization of its distribution. After entry into tumor tissues, the nanoplatform was disassembled and the photoactivity of ZnPc was recovered to emit red fluorescence and generate ROS for self-reporting and PDT, respectively (Figure 7d). Furthermore, as shown in Figure 7e, the generated ROS reacted with TPCB, and TPCB was activated to emit an orange-red light for the monitoring of ROS release and the PDT effect. In addition to organic materials, Song and co-workers designed a bis-metal Gd/Cu-nanosheet with activatable fluorescence for precise PDT [63]. They found that the fluorescence emission intensity of Gd/Cu nanosheets was linearly correlated with ^1^O_2_ production as well as PDT efficiency. 

**Figure 8:**
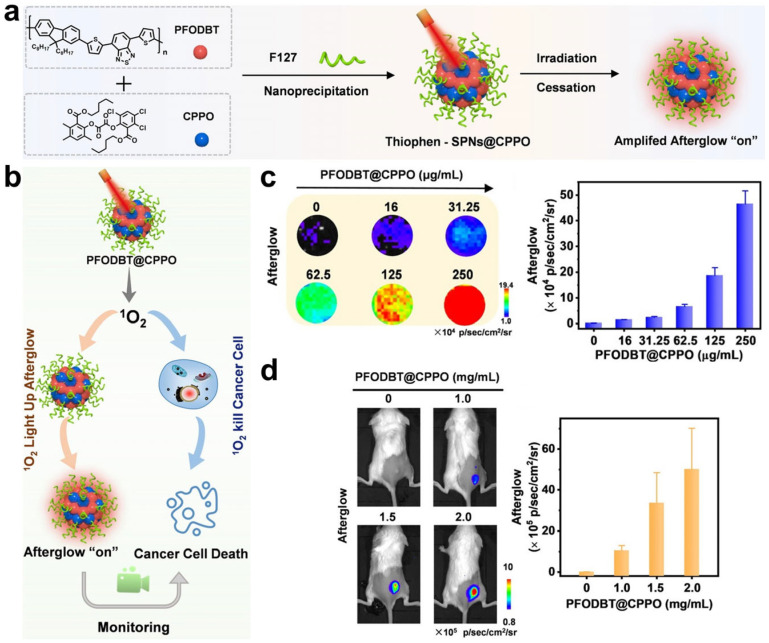
(**a**) Schematic illustration of the construction and structure of PFODBT@CPPO. (**b**) Monitoring of ^1^O_2_ generation based on persistent luminescence in PDT. Persistent luminescence images and corresponding luminescence intensity of ^1^O_2_ generation in (**c**) cancer cells and (**d**) tumor-bearing mice treated with PFODBT@CPPO. Reprinted with permission from ref. [64]. Copyright 2021, WILEY-VCH.

To avoid tissue auto-fluorescence interference caused by in situ light illumination, persistent luminescence and chemiluminescence probes responding to ROS were developed to improve the imaging sensitivity and signal-to-background ratio (SBR) during PDT. For example, an organic persistent luminescence nanoreporter PFODBT@CPPO was synthesized by Zhang et al., which can perform the self-generation and self-monitoring of ROS (Figure 8a) [64]. The semiconducting polymer (PFODBT) acts as a PS and NIR probe to generate and detect ^1^O_2_, and the chemiluminescent substrate (CPPO) is used to facilitate ^1^O_2_ production and improve persistent luminescence intensity (Figure 8b). The produced ^1^O_2_ killed cancer cells and further induce an immune responses to suppress tumor growth, and ^1^O_2_ production and therapeutic performance were well-correlated with persistent luminescent signals (Figure 8c,d). This nanoreporter provides a potential tool for the prediction of anti-cancer efficiency in PDT. Recently, another organic persistent luminescence nanoreporter based on donor–acceptor conjugated copolymers was designed with an NIR emission wavelength over 800 nm [65]. This nanoreporter achieved the real-time persistent luminescence imaging of ^1^O_2_ and cancer inhibition rate with high signal-to-noise ratios.

**Figure 9:**
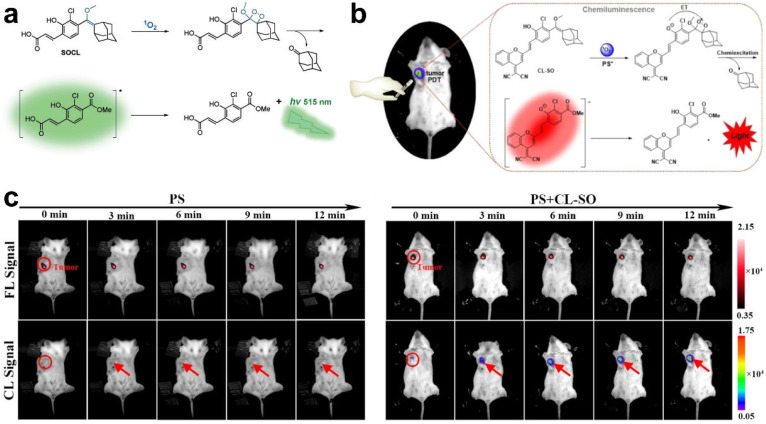
(**a**) Structure and ^1^O_2_-detection mechanism of chemiluminescent probe SOCL. Reprinted with permission from ref. [58]. Copyright 2017, WILEY-VCH. (**b**) Chemiluminescent pathway of CL-SO upon reaction with ^1^O_2_. (**c**) Fluorescence and chemiluminescence images for monitoring PS and ^1^O_2_ generation during PDT. Reprinted with permission from ref. [66]. Copyright 2020. American Chemistry Society.

Chemiluminescence probes were also developed to detect ^1^O_2_ with high selectivity and sensitivity. In Shabat’s work, a chemiluminescence probe (SOCL-CPP) was designed to visualize ^1^O_2_ production in living cells [58]. After reacting with ^1^O_2_, SOCL-CPP formed a dioxetane and decomposed through a chemiexcitation pathway, resulting in a strong green-light emission (Figure 9a). HeLa cells were treated with PSs and intracellular ^1^O_2_ production was monitored by measuring the change in the light emission intensity of SOCL-CPP. Three years later, the same group created an NIR chemiluminescent probe (CL-SO) by caging a phenoxy-dioxetane precursor scaffold and a dicyanomethylchromone acceptor to monitor ^1^O_2_ in living cells and mice [66]. As shown in Figure 9b, CL-SO is oxidized by ^1^O_2_ to a phenol-dioxetane species and disaggregates to a carbonyl structure in an excited state, leading to NIR chemiluminescence production through decay from the excited state to the ground state. The ^1^O_2_ generated by PSs in cancer cells and tumors of mice during the PDT process was effectively detected by imaging the turn-on chemiluminescence signal of CL-SO (Figure 9c). The abovementioned examples suggest that fluorescence probes, including photoluminescence, persistent luminescence, and chemiluminescence, can be well-designed to monitor ^1^O_2_ generation in PDT and predict anti-tumor effects, as well as provide precise guidance for PDT.

### 4.3. Monitoring of Cell Apoptosis

**Figure 10:**
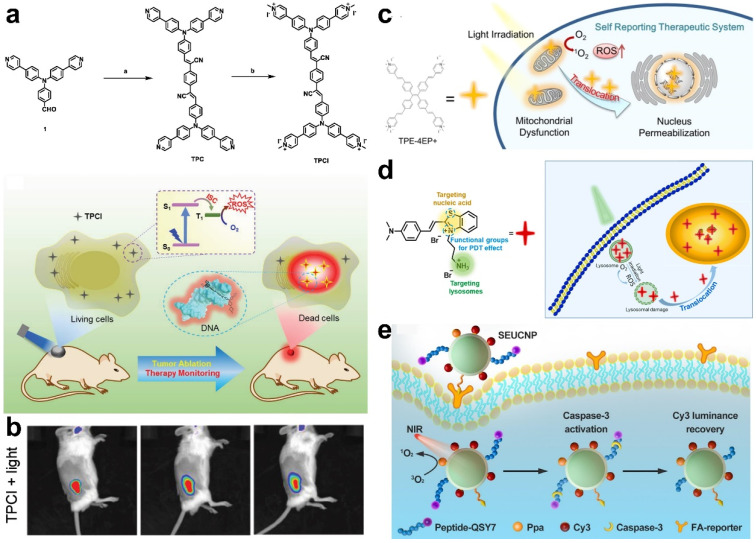
(**a**) Synthetic route of TPCI and the corresponding apoptosis-monitoring mechanism. (**b**) Fluorescence imaging of cell apoptosis in tumor-bearing mice after PDT treatment. Reprinted with permission from ref. [68]. Copyright 2019, WILEY-VCH. (**c**) Self-monitoring of apoptosis based on mitochondria-to-nucleus translocation of TPE-4EP+. Reprinted with permission from ref. [69]. Copyright 2019. American Chemistry Society. (**d**) Structure and mechanism of NSLN to report apoptosis based on lysosomes-to-nucleus translocation. Reprinted with permission from ref. [71]. Copyright 2023. Elsevier B. V. (**e**) Schematic illustration of LRET-based UCNPs for caspase-3 imaging during PDT [72].

It is generally recognized that PDT can induce the apoptosis of cancer cells due to the excessive accumulation of ROS [30]. The real-time monitoring of cell apoptosis can provide valuable information about the therapeutic outcomes. During the apoptosis process, cells undergo an increase in the cell membrane and karyotheca permeability, depolarization of the mitochondrial membrane, lysosome damage, DNA fragmentation, apoptosis-marker caspase-3 production, etc. [67]. Based on these phenomena, some probes sensing apoptosis were reported to provide real-time information on PDT effects. In Tang’s work, a dual-functional AIE molecule, TPCI, was synthesized, which shows an exceptional ^1^O_2_ quantum yield and can self-report PDT outcomes (Figure 10a) [68]. TPCI processed a high ΦΔ (98.6%) and low-fluorescence quantum yield (Φ_f_) (0.002), respectively, indicating an excellent PDT performance and suppressed fluorescence emissions. However, upon the addition of DNA, TPCI bound with DNA and aggregated, leading to the enhancement of fluorescence and a decrease in ^1^O_2_ generation. As demonstrated by the experiments, Φ_f_ of TPCI increased 15-fold to 0.03 and ΦΔ of TPCI decreased to a value of 7.0%, which is crucial to monitor PDT efficiency. After being taken up by cancer cells, TPCI could hardly penetrate karyotheca and exhibited negligible fluorescence. Upon ^1^O_2_-mediated PDT, cancer cells were killed and the permeability of karyotheca changed. On account of its high binding affinity to nuclear DNA, TPCI translocated across karyotheca and bound to nuclear DNA with an aggregative state, resulting in the emission of AIE fluorescence in the cell nucleus. In vivo experiments demonstrated that TPCI not only induced cell ablation, but also self-monitored the apoptosis of cancer cells to reflect the therapeutic effects (Figure 10b). The same group designed another AIE molecule, TPE-4EP+, to monitor cell apoptosis based on the mitochondria-to-nucleus translocation induced by apoptosis [69]. As shown in Figure 10c, after endocytosis by cancer cells, positively charged TPE-4EP+ targets the negatively charged mitochondrial membrane and enters the mitochondria. Upon light irradiation, TPE-4EP+ produced ^1^O_2_ and evoked cell ablation, along with mitochondrial depolarization, karyotheca permeabilization, and the degradation of nuclear DNA. As a result, TPE-4EP+ translocated to the nucleus owing to the electrostatic interaction between nuclear DNA and TPE-4EP+. Thus, the PDT-mediated apoptosis process was visualized by imaging the fluorescence translocation signal from mitochondria to the nucleus. One year later, Li and co-workers reported another AIE molecule, TTVPHE, which could generate ROS to trigger PDT and achieve the self-monitoring of the PDT process through the apoptosis-induced translocation from mitochondria to the nucleus membrane [70]. 

Recently, Yu et al. reported a lysosome-targeted and self-monitoring probe, NSLN, based on lysosome-to-nucleus immigration (Figure 10d) [72]. Initially, NSLN stained lysosomes in cancer cells, but immigrated into the nucleus through binding with DNA after PDT-induced apoptosis and lysosome damage. The cell apoptosis process in PDT was monitored in real time by observing the subcellular immigration of the NSLN signal.

Caspase-3, as a cell apoptosis marker, was monitored by Ju et al. to evaluate the PDT effect using luminescence resonance energy transfer (LRET)-based UCNPs (Figure 10e) [72]. Dye Cy3 and PS Ppa as an energy collector and QSY7-labeled peptide were functionalized on the surface of UCNPs. The emitted energy from UCNPs was absorbed by Cy3 and Ppa, then the energy of Cy3 was further pumped to QSY7. As a result, the fluorescence of Cy3 was quenched and ROS was produced for PDT. Subsequently, caspase-3 was produced to cleave the peptide, releasing QSY7 to turn on the fluorescence of Cy3. The real-time monitoring of PDT effects was realized by visualizing the “off–on” fluorescence signal of Cy3. The abovementioned works provide strategies for monitoring the PDT process based on cell apoptosis, and show great potential in the development of precise PDT.

### 4.4. Monitoring of Other Biomarkers

**Figure 11:**
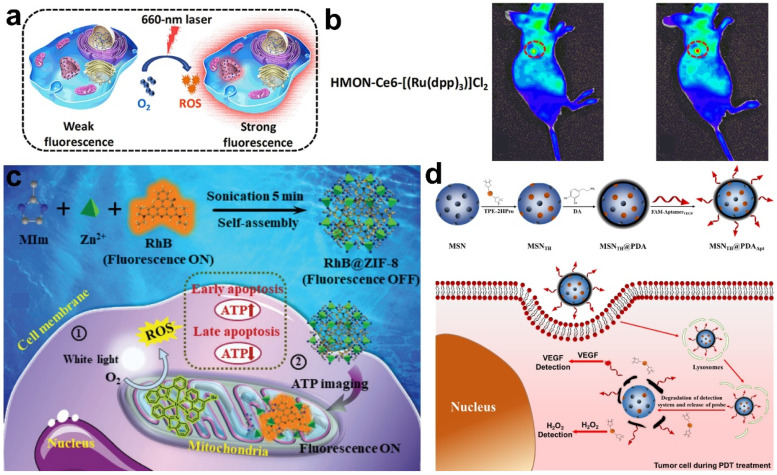
(**a**) Schematic illustration of the real-time monitoring of oxygen consumption during PDT. (**b**) The optical images of tumor-bearing mice treated with HMON-Ce6-[(Ru(dpp)_3_)]Cl_2_ before (**left**) and after (**right**) laser exposure. Reprinted with permission from ref. [74]. Copyright 2019. American Chemistry Society. (**c**) Synthesis route and ATP-monitoring mechanism during PDT based on rhodamine B@ZIF-8. Reprinted with permission from ref. [75]. Copyright 2020. The Royal Society of Chemistry. (**d**) The construction of MSN_TH_@PDA_Apt_ and its detection mechanism towards H_2_O_2_ and VEGF during PDT. Reprinted with permission from ref. [76]. Copyright 2023. American Chemistry Society.

The oxygen molecule is an important element during the PDT process, which absorbs the energy from PS and promotes the generation of ROS [31]. The oxygen concentration plays a decisive role in PDT and severely affects PDT effects. Thus, the real-time visualization of oxygen change is crucial to predict therapeutic outcomes and to optimize PDT protocols. In Zhang’s work, PS chlorin e6 (Ce6) and oxygen probe [(Ru(dpp)_3_)]Cl_2_ were embedded into hollow mesoporous organosilica nanoparticles (HMONs) (named HMON-Ce6-[(Ru(dpp)_3_)]Cl_2_) for the simultaneous PDT and self-monitoring of oxygen consumption [73]. Initially, the high level of oxygen quenched the fluorescence of [(Ru(dpp)_3_)]Cl_2_, and this nanoprobe showed negligible red fluorescence. Under light irradiation, the level of oxygen decreased due to the consumption of oxygen to produce ROS, leading to the strengthening of the red fluorescence signal (Figure 11a). In vitro tests showed that this nanoprobe could realize the detection of 1% to 20% oxygen changes. Furthermore, the in vivo experiment results suggest the ability of this nanoprobe to monitor oxygen consumption through the off–on fluorescence of [(Ru(dpp)_3_)]Cl_2_ (Figure 11b).

ATP is the major energy source in organisms and is crucial to many cellular processes, such as metabolism and apoptosis [74]. As previously reported, decreasing the content of intracellular ATP can enhance cancer cell chemosensitivity and further improve anti-tumor efficacy. Therefore, the real-time reporting of intracellular ATP change is a potential method to evaluate PDT effects. Zhang et al. designed an ATP-responsive rhodamine B@ZIF-8 nanoprobe for monitoring ATP fluctuations in cancer cells during PDT (Figure 11c) [75]. The fluorescence of rhodamine B was quenched owing to aggregation-induced quenching, but was restored in the presence of ATP through a competitive coordination interaction and the disintegration of ZIF-8. The ATP levels were found to increase within 1 min of light-induced PDT, but decreased during the persistent PDT process, suggesting the occurrence of cell apoptosis. This work demonstrated the real-time monitoring of the fluctuations of mitochondrial ATP in living cells, which was of significance for predicting the therapeutic outcomes and understanding the function of ATP during the PDT process.

Vascular endothelial growth factor (VEGF) is an important mediator in tumor angiogenesis [77]. Tumor hypoxia upregulates VEGF expression, leading to tumor metastasis and therapy failure [78]. When intracellular oxygen is sufficient, the level of H_2_O_2_ (a kind of ROS) increases, whereas the VEGF level decreases, indicating the successful treatment of tumor cells. Thus, it is necessary to monitor the changes in VEGF and H_2_O_2_ during PDT treatment, providing guides to improve PDT efficiency. Xing et al. constructed a dual-fluorescent probe (MSN_TH_@PDA_Apt_) to detect VEGF and H_2_O_2_, which was obtained by loading the H_2_O_2_-detection probe, TPE-2HPro (AIE molecule), and VEGF probe, FAM-Aptamer_VEGF_, on polydopamine-coated mesoporous silica nanoparticles (MSN@PDA) (Figure 11d) [76]. H_2_O_2_ could react with the pinacol boronate group in TPE-2HPro and lead to its disassembly. Thus, the fluorescence of TPE-2HPro was turned on to achieve the specific detection of H_2_O_2_. In addition, the fluorescence of the VEGF probe FAM-Aptamer_VEGF_ was quenched by polydopamine via the π–π conjugation. In the presence of VEGF, FAM-Aptamer_VEGF_ was separated from polydopamine due to the specific binding to VEGF, and the fluorescence of FAM was restored, thereby realizing the monitoring of intracellular VEGF. During PDT treatment mediated by low Ce6 concentrations, the increasing level of VEGF was observed, suggesting that cancer cells were inclined to metastasis rather than death. After increasing the Ce6 concentration, the level of H_2_O_2_ was enhanced but the VEGF level gradually decreased, suggesting efficient cell ablation. The real-time monitoring of H_2_O_2_ and VEGF during PDT treatment was successfully realized in this work, which is crucial to evaluate tumor development trends and to enhance the therapeutic effect.

## 5. PTT Evaluation

The monitoring of PTT agent release and concentration, temperature increase, and cell apoptosis-related makers in tumors can maximize the effect of PTT while decreasing the undesired side effects to realize precision medicine. At present, a number of PTT agents with optical properties that are sensitive to temperature changes and apoptosis-related makers have been synthesized to provide real-time optical feedback during PTT. The monitoring performance of materials with different optical properties is systematically introduced in the following section. 

### 5.1. Monitoring of PTT Agents

**Figure 12:**
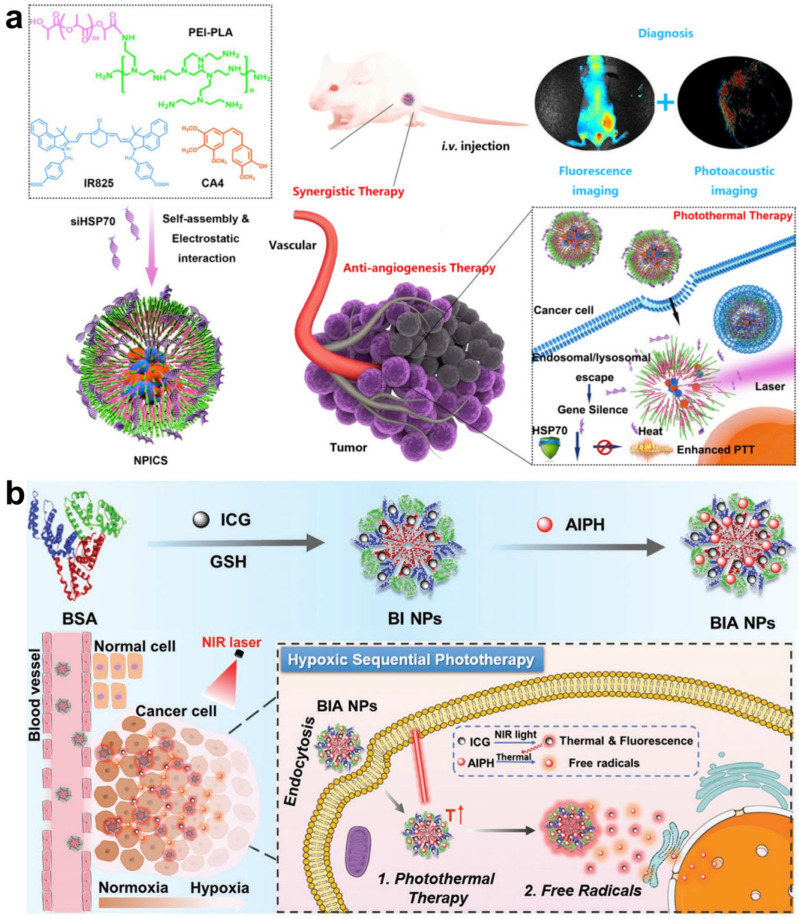
(**a**) Schematic illustration of NPICS used for synergistic therapy and self-monitoring in breast tumor-bearing mice. Reprinted with permission from ref. [79]. Copyright 2019. Elsevier B. V. (**b**) Synthesis route of BIA nanoplatform for synergistic PDT and PTT. Reprinted with permission from ref. [80]. Copyright 2019, WILEY-VCH.

Similar to the PDT process, the -time monitoring of the release and tumor accumulation of PTT agents during treatment is valuable in the enhancement of therapeutic accuracy. In Wu’s work, a triple-collaborative nanoplatform was developed for both the self-monitoring of PTT agent releases and synergistic therapy of anti-angiogenesis, PTT, and RNA interference (Figure 12a) [79]. This nanoplatform (denoted as NPICS) was constructed through the self-assembly of a polymer polyethyleneimine-polylactide (PEI-PLA) carrier, anti-angiogenesis agent combretastatin A4 (CA4), NIR dye IR825, and heat-shock protein 70 (HSP70)-inhibitor siRNA. With the synergistic effect of the CA4-induced inhibition of tumor angiogenesis and restrain of HSP70 expression, the efficacy of IR825-mediated PTT was significantly improved upon NIR laser irradiation. In addition, benefiting from the auto-fluorescence property of PEI-PLA, the distribution and accumulation of NPICS in tumors were effectively visualized in real time by fluorescence imaging. Furthermore, PTT agents IR825 in NPICS could also act as PA agents for PA imaging. This nanoplatform with fluorescence and PA dual-channel imaging showed great potential for precise tumor therapy. In the same year, a biocompatible nanoplatform was synthesized by Lee et al. for synergistic PDT and PTT, as well as the real-time monitoring of PTT agent release [80]. As shown in Figure 12b, this nanoplatform was constructed by the combination of bovine serum albumin (BSA), ICG, and a radical generator (AIPH) named BIA. Under laser irradiation, the PTT agent ICG produced heat and triggered the dissociation of AIPH to release cytotoxic alkyl radicals for cell ablation. Subsequently, the fluorescence of ICG quenched by aggregation-induced quenching was recovered, along with the release of ICG from AIPH, achieving the self-monitoring of the release process via off–on fluorescence imaging. These works highlight the importance of the real-time monitoring of PTT agents for high-performance cancer therapy.

**Figure 13:**
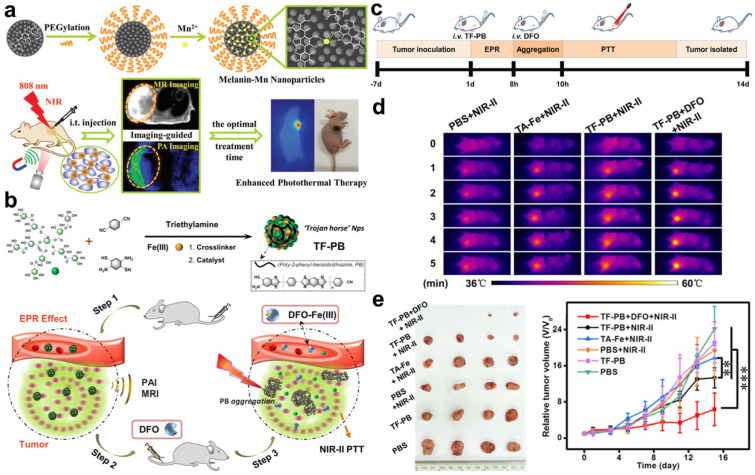
(**a**) Schematic illustration of the fabrication process of melanin-Mn nanoplatform for MR/PA dual-modality imaging-guided PTT. Reprinted with permission from ref. [81]. Copyright 2018. The Royal Society of Chemistry. (**b**) Synthesis route of TF-PB for enhanced PTT. (**c**) Schematic illustration of TF-PB-based therapy. (**d**) Imaging of temperature variations and (**e**) tumor volume of 4T1 tumor-bearing mice after the treatments of TF-PB. Reprinted with permission from ref. [82]. Copyright 2023. American Chemistry Society. Statistical difference was considered significant at a value of ** *p* < 0.01, *** *p* < 0.001, respectively.

Compared with fluorescence imaging, PA imaging possessing a high-penetration depth and spatial resolution has been demonstrated to be a promising tool for tissue imaging to provide precise guidance for cancer therapy [45]. More importantly, many PTT agents show excellent PA properties, which can act as PA agents for self-monitoring their distribution via PA imaging. Zhang and co-workers developed a multifunctional nanoplatform based on Mn^2+^ chelated endogenous biopolymer melanin for self-monitoring PTT via magnetic resonance (MR)/PA dual-modality imaging (Figure 13a) [81]. In vivo dual-modality imaging experiments found that the nanoplatform first diffused in the blood vessel upon intratumoral injection, and then entered the whole tumors after 3 h. The subsequent PTT results showed an excellent tumor-suppressor performance with imaging guidance. Therefore, the real-time monitoring of the distribution of nanoplatforms was crucial for determining the optimal treatment time, enhancing the therapeutic accuracy and effect. To maximize the tumor accumulation and retention of nanoplatforms, the same group reported an artificially controllable nanoplatform, TF-PB, to enhance the imaging accuracy and PTT effect by prolonging the tumor retention time [82]. Hydrophobic poly-2-phenylbenzobisthiazole (PB) with photothermal properties was combined with a hydrophilic polyphenol-Fe(III) structure to construct TF-PB that showed PA and MR properties. Once artificially injected with deferoxamine (DFO), chelation between DFO and Fe(III) occurred and destroyed the structure of TF-PB, leading to the release and hydrophobic aggregation of PB. As a result, the aggregated PB with a longer tumor retention time and stronger PA signal achieved the accurate real-time monitoring of nanoplatform accumulation and enhanced PTT effect, which was demonstrated in 4T1 tumor-bearing mice (Figure 13c–e). Zhang’s work suggests that the self-monitoring of PTT agents based on PA imaging is of great significance for highly efficient and precise PTT.

### 5.2. Monitoring of Temperature

**Figure 14:**
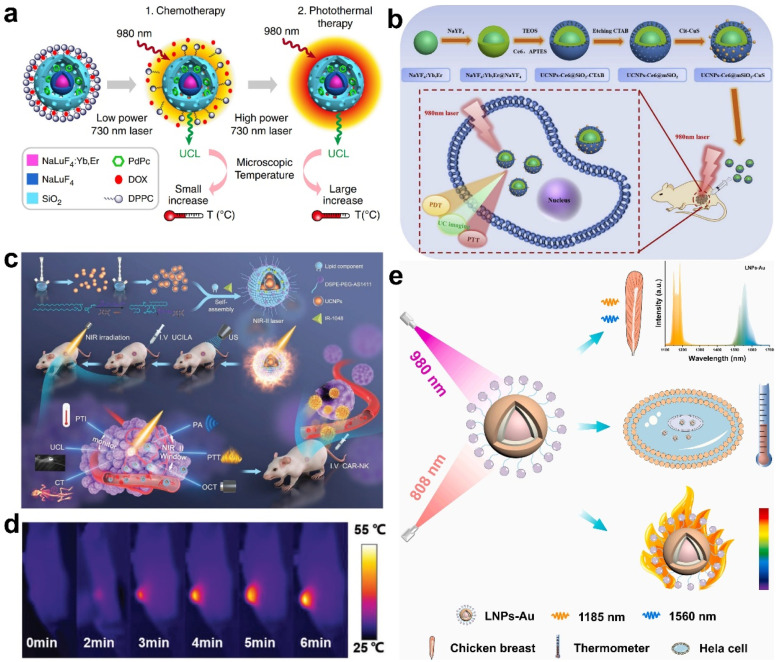
(**a**) Schematic illustration of programmed synergistic chemotherapy and PTT [85]. (**b**) Synthesis route of UCNPs-Ce6@mSiO_2_-CuS nanoplatform and the temperature-monitored synergistic PDT and PTT. Reprinted with permission from ref. [85]. Copyright 2019. Elsevier B. V. (**c**) Schematic illustration of penta-modal imaging-guided and temperature-feedback PTT and CAR-NK immunotherapy based on UCILA nanoplatform. (**d**) In vivo photothermal images of temperature changes in the tumor site. Reprinted with permission from ref. [86]. Copyright 2021, WILEY-VCH. (**e**) Schematic illustration of real-time temperature measurement using LNPs-Au nanoplatform. Reprinted with permission from ref. [87]. Copyright 2023. Elsevier B. V.

It is known that the suitable temperature for cancer cell ablation is 42–45 °C [83]. Temperatures above 45 °C may induce damage to surrounding normal tissues, while cancer cells cannot be completely killed when the temperature is below 42 °C. Therefore, accurate determination of temperature changes during PTT can help control and optimize PDT treatment parameters to increase therapy safety and alleviate the adverse consequences. To date, some optical probes responding to temperature changes, such as UCNPs, organic dyes, polymers, bioactive glasses, quantum dots, and molecular beacons, have been proposed to provide temperature feedback during PTT in real time. The temperature-responsive luminescence of UCNPs makes them a promising candidate for the monitoring of temperature. For example, Li’s group reported a temperature-responsive nanoplatform based on UCNPs for synergistic chemotherapy and PTT [84]. This nanoplatform was obtained through the combination of UCNPs, PTT agent (PdPc), SiO_2_, chemotherapeutic drug (Dox), and thermal responsive drug release unit (DPPC micelle), denoted as TR-UCNS. The PTT effect of TR-UCNS was triggered by the excitation of a 730 nm laser, while the temperature during PTT was monitored by recording the temperature-responsive luminescence of UCNPs upon irradiation of a 980 nm laser. As shown in Figure 14a, the temperature of TR-UCNS could be well-tuned by adjusting the power density of the 730 nm laser to furtherly induce Dox release and PTT stepwise. The sequence of chemotherapy and PTT was monitored through luminescence-based temperature feedback. Cell experiments proved that chemotherapy administered before PTT showed better therapeutic outcomes. This programmed strategy presented in this work was demonstrated to achieve optimal synergistic chemotherapy and PTT with the assistance of temperature monitoring. With a similar design, PDT and PTT combination therapy was realized by Song et al., in which UCNPs also served as temperature reporters [85]. Mesoporous SiO_2_ was coated on UCNPs and the nanoparticles were further modified with Ce6 to absorb the red emission of UCNPs for PDT. Finally, PTT agent Cit-CuS nanoparticles were linked with the composite to acquire the UCNPs-Ce6@mSiO_2_-CuS nanoplatform (Figure 14b). The temperature was real-time monitored by observing the ratio of I_525_/I_545_ of UCNPs emissions during therapy, and the temperature operating curve was obtained in both cellular and animal experiments. 

With the purpose of improving the accuracy and efficacy of therapy, Yuan’s group proposed a multifunctional nanoplatform for penta-modal imaging and temperature-monitored PTT (Figure 14c) [86]. The nanoplatform UCILA was self-assembled with UCNPs, IR-1048 dye, and lipid–aptamer, in which IR-1048 was applied for PA, optical coherence tomography angiography, and photothermal imaging, as well as the PTT agent. Additionally, UCNPs with high X-ray attenuation coefficients and upconversion luminescence can serve as computed tomography contrast agents and temperature-responsive probes. Benefiting from the self-reporting of temperature and guidance of penta-modal imaging (Figure 14d), UCILA showed a robust tumor-suppressor capability toward lung tumors with negligible side effects. Furthermore, chimeric antigen receptor-modified natural killer (CAR-NK) cells were complementally injected after PTT to perform synergistic immunotherapy for complete cell ablation. The luminescence of lanthanide nanoparticles (LNPs) can be extended to the NIR-II region through doping with rare earth cations, such as Er^3+^, Ce^3+^, Ho^3+^, and Yb^3+^, for the complete elimination of interferences from tissues and light scattering. In Xu’s work, an LNPs-gold (LNPs-Au) nanoplatform was reported for temperature feedback in PTT [87]. The emission wavelength at 1185 and 1560 nm of LNPs-Au is sensitive to temperature, which is suitable to monitor temperature changes during Au-mediated PTT (Figure 14e). These examples demonstrate the great performance of UCNPs for temperature measurements, which will motivate the development of precise PTT and synergistic therapy.

**Figure 15:**
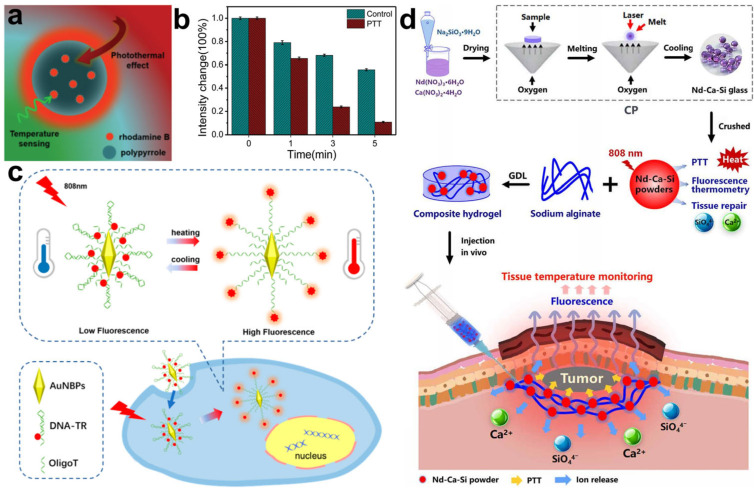
(**a**) Schematic illustration of PPy–rhodamine B nanoplatform for PTT and temperature sensing. (**b**) Fluorescence signal changes in cells treated with and without PTT. Reprinted with permission from ref. [88]. Copyright 2020. The Royal Society of Chemistry. (**c**) Schematic illustration of DNA stem-loop-based nanoplatform for PTT and temperature monitoring. Reprinted with permission from ref. [89]. Copyright 2019. American Chemistry Society. (**d**) Synthesis route of Nd-Ca-Si bioactive glass/alginate composite hydrogel and its application for temperature-monitored PTT and tissue repair [90].

In addition to UCNPs, organic dye can also serve as a temperature-sensing probe. Yang et al. combined polypyrrole (PPy) and rhodamine B via polymerization to construct a dual-functional nanoplatform to provide temperature feedback in PTT (Figure 15a) [88]. In this nanoplatform, PPy acted as PTT agent and rhodamine B was responsible for monitoring the temperature changes during therapy by producing a temperature-dependent fluorescence intensity. Using HepG2 cells as models, the PPy–rhodamine B nanoplatform was demonstrated to show a reliable cell ablation performance as well as the capability of self-monitoring PTT processes (Figure 15b). Molecular beacons sensitive to temperature can also be used to construct probes to monitor the temperature changes in PTT. In Shi’s work, a Texas Red and gold nanobipyramid were linked at two ends of the temperature-sensitive DNA stem-loop, respectively, to prepare a nanoplatform that performed PTT and temperature monitoring (Figure 15c) [89]. Gold nanobipyramids can realize photothermal conversions and quench the fluorescence of Texas Red. Upon laser irradiation, the temperature was elevated due to the photothermal effect of the gold nanobipyramid, resulting in the opening of the DNA stem-loop and the accompanied separation of Texas Red from the gold nanobipyramid. Subsequently, the temperature change during PTT was visualized along with the recovery of Texas Red fluorescence. The fluorescence of Texas Red was significantly enhanced in cellular experiments under 808 nm light illumination, suggesting that the cancer cells were efficiently killed after treatment with this nanoplatform. Bioactive glasses play crucial roles in tissue regeneration and wound healing. Nd was doped into bioactive glasses by Chang’s group, which wedded the glasses with temperature-sensitive fluorescence properties and photothermal conversion ability [90]. As shown in Figure 15d, an Nd-Ca-Si bioactive glass/alginate composite hydrogel was synthesized for temperature-monitored PTT and tissue repair. The fluorescence intensity of the hydrogel was found to be linearly correlated with the temperature changes in wounds illuminated by an 808 nm laser. Furthermore, the tissue damage caused by overheating in PTT was repaired by this bioactive hydrogel. Owning to the temperature-monitoring function and wound healing bioactivity, the constructed bioactive glass hydrogel exhibited effective and reliable tumor treatment without damage to normal tissue. These works indicate that the fluorescence of UCNPs, dyes, and bioactive glass can be applied as thermal feedback to monitor the PTT process.

**Figure 16:**
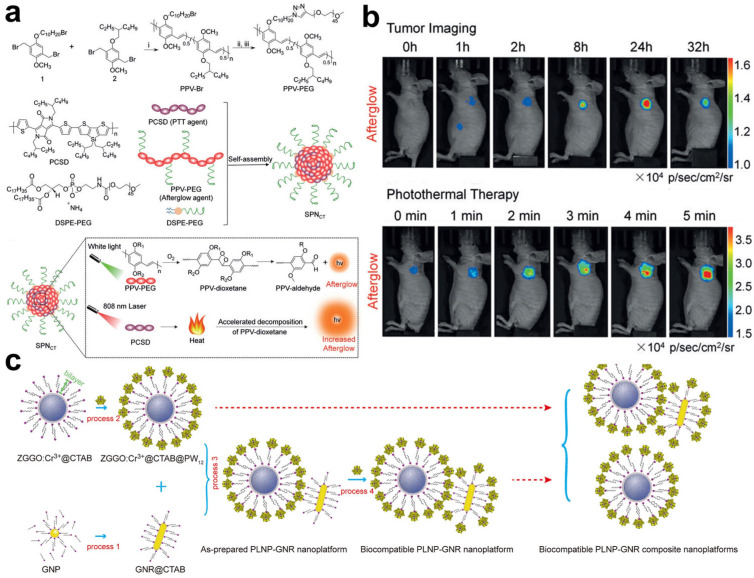
(**a**) Synthetic routes of SPN_CT_ and the mechanism of temperature-dependent persistent luminescence. (**b**) In vivo imaging of 4T1 tumor-bearing mice at different times after injection of SPN_CT_ (above) and persistent luminescence imaging of tumor for temperature monitoring under laser irradiation for different times after 24 h of injection. Reprinted with permission from ref. [91]. Copyright 2018, WILEY-VCH. (**c**) Synthetic procedures of PLNP-GNR nanoplatform. Reprinted with permission from ref. [92]. Copyright 2021. Elsevier B. V.

Persistent luminescence nanomaterials provide a higher signal-to-background ratio in bioimaging than traditional organic dyes or quantum dots by avoiding tissue autofluorescence interference [47]. Pu et al. prepared a semiconducting polymer nanococktail (SPN_CT_) for imaging-guided PTT based on its temperature-dependent persistent luminescence intensity [91]. SPN_CT_ was synthesized through the hydrophobic interaction between amphiphilic poly(ethylene glycol) (PEG) grafted poly(phenylenevinylene) (PPV) and hydrophobic poly(silolodithiophene-alt-diketopyrrolopyrrole (PCSD) (Figure 16a). In SPN_CT_, PPV acted as the temperature-reporting probe to produce temperature-related persistent luminescence based on the thermodynamically controllable decomposition of dioxetane units in PPV. Meanwhile, PCSD within SPN_CT_ absorbed the NIR light to generate heat for PTT (Figure 16a). Once intravenously injected, the distribution and accumulation of SPN_CT_ in tumors were visualized via persistent luminescence imaging (Figure 16b). Under the irradiation of an NIR laser, PCSD produced heat to increase the temperature in tumors. Subsequently, the persistent luminescence of SPN_CT_ was enhanced to report the photothermal temperature of the PTT process (Figure 16b). The SPN_CT_ presented in this work achieved real-time temperature-monitoring PTT without the interference of tissue autofluorescence because real-time light excitation was not involved. In addition to organic semiconducting persistent luminescent molecules, inorganic persistent luminescent nanoparticles (PLNPs) have also been widely applied in bioimaging. Liu and coworkers modified hexadecyl trimethyl ammonium bromide (CTAB) and phosphotungstic acid (PW12) molecules on the surface of ZGGO:Cr^3+^ PLNPs and gold nanorods (GNRs) for the self-assembly of PLNPs and GNRs (Figure 16c) [92]. The resultant PLNP-GNR nanoplatform was able to monitor the temperature changes during PTT via autofluorescence-free bioimaging. In this PLNP-GNR nanoplatform, PLNPs served as an optical sensor for temperature measurements due to their temperature sensitivities. The thermosensitive property of PLNPs mainly originated from Cr^3+^. With the increase in temperature, the luminescence increases in interior Cr^3+^ were higher than that of surface Cr^3+^ at 35.7–47.9 °C. Based on these works, we can imagine that persistent luminescent nanomaterials have potential applications for autofluorescence-free imaging-guided PTT to realize precision medicine.

### 5.3. Monitoring of Cell Apoptosis

**Figure 17:**
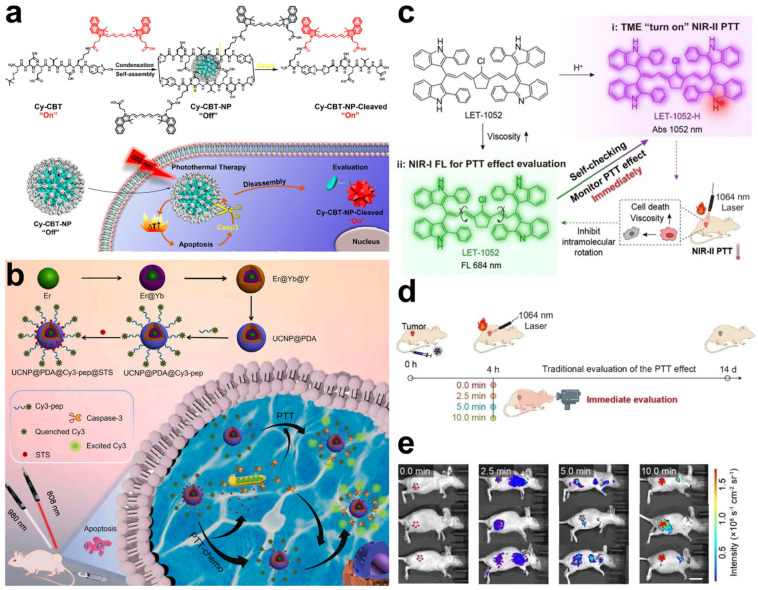
(**a**) Structure, self-assembly process, and caspase-3-responsive mechanism of Cy−CBT nanoparticles for apoptosis monitoring. Reprinted with permission from ref. [95]. Copyright 2020. American Chemistry Society. (**b**) Schematic illustration of constructing UCNP@polydopamine@Cy3−pepitide nanoparticles and their application in real-time caspase-3 monitoring during synergistical PTT and chemotherapy. Reprinted with permission from ref. [96]. Copyright 2021. Elsevier B. V. (**c**) Schematic illustration of pH/viscosity activatable LET-1052 for in situ activation and real-time monitoring of PTT efficacy based on viscosity. (**d**) Schematic illustration of evaluation of PTT effect based on fluorescence imaging. (**e**) In vivo NIR fluorescence imaging of LET-1052 with different laser irradiation times in 4T1 tumor-bearing mice treated with LET-1052. Reprinted with permission from ref. [99]. Copyright 2022, WILEY-VCH.

Similar to PDT, the apoptosis of cancer cells is induced by high temperatures in PTT. Caspase-3 plays an important role during cell apoptosis and the activity of caspase-3 is considered a promising biomarker to evaluate the therapeutic effect of PTT [93]. A specific peptide substrate of caspase-3 (Asp-Glu-Val-Asp, denoted as DEVD) is usually employed for constructing apoptosis-sensitive probes [94]. In Liang’s work, a precursor Cys(StBu)-DEVD-Lys(Cypate)-CBT (namely, Cy-CBT) was prepared through a CBT-Cys click condensation reaction, in which Cypate exhibited an NIR fluorescence emission and potent photothermal conversion properties [95]. Furthermore, due to the disulfide bond reduction, Cy-CBT was self-assembled into a nonfluorescent nanoparticle, owing to intra- and intermolecular quenching effects (Figure 17a). Once PTT-mediated cell apoptosis occurred, caspase-3 was generated to degrade DEVD substrates, leading to the disintegration of Cy-CBT nanoparticles. As a result, the fluorescence of Cypate was recovered to realize the monitoring of cell apoptosis, as well as enhance the PTT effects (Figure 17a). In vivo experiments also proved that the “turn-on” fluorescence of Cy-CBT nanoparticles was successfully used to evaluate PTT efficacy. One year later, another caspase-3-responsive multifunctional nanoparticle based on DEVD substrates was reported by Song et al. [96]. Polydopamine was coated on the surface of UCNPs to serve as a quencher and PTT agent; then, chemotherapeutic drug staurosporine (STS) and Cy3-labeled DEVD were loaded onto the polydopamine surface (Figure 17b). FRET occurred between the polydopamine and Cy3; however, the fluorescence of Cy3 was restored in the presence of caspase-3. Using the luminescence of UCNPs as an internal reference, the ratio of Cy3 fluorescence to upconversion luminescence was linearly correlated with caspase-3 activity. In vitro tests demonstrated that this nanoparticle detected caspase-3 in the concentration range of 0.5–50 ng/mL, with a low limit of detection at 0.065 ng/mL. In addition, the nanoparticles successfully achieved the real-time evaluation of cell apoptosis during synergistic PTT and chemotherapy. 

In addition to caspase-3, an abnormal increase in intracellular viscosity is also a considerable physiological feature of cell apoptosis [97]. Viscosity in cells is related to many cellular processes, such as protein aggregation, signal transduction, and cell apoptosis [98]. Especially during cell apoptosis, the cytoplasm coagulates and the cells shrink, leading to the elevation of cytoplasmic viscosity. Lin et al. designed a pH/viscosity-responsive probe, named LET-1052, by regulating the intramolecular rotation for activatable PTT [99]. Initially, the photothermal conversion ability and fluorescence emission of LET-1052 were inhibited. Once LET-1052 entered the acidic tumor microenvironment, LET-1052 was protonated, along with the activation of photothermal conversion capacity for PTT upon irradiation with a 1064 nm laser (Figure 17c). As expected, PTT-mediated cell apoptosis elevated the viscosity of the cancer cells, inducing the inhibition of intramolecular rotation and decreasing non-radiative decay. Consequently, the NIR fluorescence emission of LET-1052 was significantly enhanced for viscosity monitoring, realizing the assessment of the PTT effect in real time (Figure 17c). After the treatment of tumor-bearing mice with LET-1052, tumor growth was successfully suppressed and the NIR fluorescence intensity in the tumors was observed to be positively correlated to the tumor suppressor rate, suggesting the robust performance of LET-1052 in the prediction of PTT efficiency (Figure 17d,e). These sensing nanoparticles showed a reliable performance in the non-invasive assessment of tumor suppression, providing a potential tool for promoting the accuracy and efficacy of PTT.

## 6. Conclusions and Perspectives

In this review, we presented the advances achieved when evaluating cancer phototherapy during the past five years based on optical imaging (Table 1). In the evaluation of PDT, PS release, ROS production, cell apoptosis, tissue oxygen consumption, ATP, and other biomarkers were detected in real time to provide feedback on PDT effects. Similarly, in the monitoring of PTT, the optical intensity was altered with the release of PTT agents, temperature change, and cell apoptosis to realize the self-reporting of PTT effects. Great progress has been achieved in the development of self-reporting therapeutic agents for the real-time evaluation of cancer phototherapy. There are still many challenges faced by self-reporting therapeutic agents for safer phototherapy techniques and evaluations with higher spatial resolutions.

(1) Improving the biocompatibility of therapeutic agents. The therapeutic agents are usually composed of metal ions and a polycyclic structure, which inevitably causes unexpected damage to the skin, normal tissues, or organs during blood circulation. It is highly necessary to synthesize more safe and innoxious agents or design agents using natural drugs to obtain therapeutic agents with high biocompatibility and degradability levels for safer cancer therapy.
(2) Enhancing the specificity of therapeutic agents toward tumors. Most of the therapeutic agents display side effects due to the always-on model under light and a lack of specificity toward tumors, resulting in undesired damage to normal tissues. In order to create precise medicine, developing activatable self-reporting agents that can only be activated in the tumor microenvironment is of great significance for the improvement of therapeutic accuracy and minimization of side effects.
(3) Evaluating therapy performance based on the simultaneous imaging of multiple biomarkers. The therapy process and dynamic changes in tumors are complex. The signal produced by a single parameter may lead to a false-positive signal or limited signal–noise ratio, resulting in an incorrect evaluation of the therapy’s performance. Designing self-reporting agents for the simultaneous monitoring of multiple pathological parameters is crucial to enhance the precision of cancer therapy evaluations.
(4) Increasing the penetration depth of therapeutic agents. The limited penetration depth of visible-emitting agents hinders in vivo applications. The luminescent units in agents need to be synthesized with extended absorption and emission wavelengths to the NIR, even NIR-II, window for deep-seated tumor therapy and high-resolution imaging.

## Figures and Tables

**Table 1:** Overview of chemicals used for phototherapy evolution based on various optical imaging technologies.

Optical Imaging Technology	Chemicals	Biomarkers	Phototherapy Evolution	Ref.
Photoluminescence imaging	Organic PS AlPcSNa_4_	PS release	PDT	[55]
	ZnPc@Cur-S-OA self-assembly nanoparticles	PS release	PDT	[56]
	Squaric acid–coumarin–chlorambucil (Sq-Cou-Cbl) nanoconjugates	PS release	PDT	[100]
	FRET-based UCNPs nanoplatform	ROS, caspase-3	PDT	[59,60,72]
	Protoporphyrin IX, 2-pyridone, and Cy7 embedded in silica nanocarrier	ROS	PDT	[61]
	ZnPc@TPCB nanoparticles	ROS	PDT	[62]
	Gd/Cu nanosheets	ROS	PDT	[63]
	TCPP@DPA-MOF-Pt nanoplatform	ROS	PDT	[101]
	AIE molecule TPCI, TPE-4EP+, TTVPHE, TIdBO, and TPA3	Cell Apoptosis	PDT	[68,69,70,102,103]
	Organic PS NSLN	Cell Apoptosis	PDT	[71]
Photoluminescence imaging	HMON-Ce6-[(Ru(dpp)_3_)]Cl_2_ nanoparticles	Oxygen	PDT	[73]
	Rhodamine B@ZIF-8 nanoprobe	ATP	PDT	[75]
	MSN_TH_@PDA_Apt_ fluorescent probe	VEGF, ROS	PDT	[76]
	Organic PS protoporphyrin IX	PS biosynthesis	PDT	[104]
	Ru-NBD probe	H_2_S	PDT	[105]
	NPICS, BIA self-assembly nanoparticles	PTT agent release	PTT	[79,80]
	TR-UCNS nanoplatform	Temperature	PTT	[84]
	UCNPs-based nanoplatform	Temperature	PTT	[85,86,106,107,108,109]
	LNPs-Au nanoplatform	Temperature	PTT	[87]
	Polypyrrole–rhodamine B polymer nanoparticles	Temperature	PTT	[88]
	Gold nanobipyramids–DNA–Texas Red nanoplatform	Temperature	PTT	[89]
	Bioactive Nd-Ca-Si glasses	Temperature	PTT	[90]
	Cy-CBT self-assembly nanoparticles	Caspase-3	PTT	[95]
	UCNP@PDA@Cy3-pep nanoplatform	Caspase-3	PTT	[96]
	Polymethine dye LET-1052	Viscosity	PTT	[99]
	RGD-CuS-Cy5.5 nanoparticle	Sentinel lymph node metastasis	PTT	[110]
Persistent luminescence imaging	PFODBT@CPPO semiconducting polymer	ROS	PDT	[64]
	Pyrido pyrazine-thiophene semiconducting polymer	ROS	PDT	[65]
	SPN_CT_ semiconducting polymer	Temperature	PTT	[91]
	ZGGO:Cr^3+^ PLNP-GNR composite nanoplatforms	Temperature	PTT	[92]
Chemiluminescence imaging	SOCL-CPP	ROS	PDT	[58]
	CL-SO	ROS	PDT	[66]
Photoacoustic imaging	Organic PS CS-P	PS release	PDT	[57]
	Mn^2+^-biopolymer melanin	PTT agent release	PTT	[81]
	TF-PB self-assembly nanoparticles	PTT agent release	PTT	[82]
	Au NRs@DSFDSs self-assembly nanoparticles	PTT agent release	PTT	[111]
	WS_2_-PEG nanosheets	Temperature	PTT	[112]

## Data Availability

Not applicable.
